# Trends and hot topics in linguistics studies from 2011 to 2021: A bibliometric analysis of highly cited papers

**DOI:** 10.3389/fpsyg.2022.1052586

**Published:** 2023-01-11

**Authors:** Sheng Yan, Le Zhang

**Affiliations:** School of Foreign Languages, Central China Normal University, Wuhan, China

**Keywords:** bibliometric analysis, linguistics, highly cited papers, corpus analysis, research trends

## Abstract

High citations most often characterize quality research that reflects the foci of the discipline. This study aims to spotlight the most recent hot topics and the trends looming from the highly cited papers (HCPs) in Web of Science category of *linguistics* and *language & linguistics* with bibliometric analysis. The bibliometric information of the 143 HCPs based on Essential Citation Indicators was retrieved and used to identify and analyze influential contributors at the levels of journals, authors, and countries. The most frequently explored topics were identified by corpus analysis and manual checking. The retrieved topics can be grouped into five general categories: *multilingual-related*, *language teaching*, and *learning related*, *psycho/pathological/cognitive linguistics-related*, *methods and tools-related*, and *others*. Topics such as *bi/multilingual(ism)*, *translanguaging*, *language/writing development*, *models*, *emotions*, *foreign language enjoyment (FLE)*, *cognition*, *anxiety* are among the most frequently explored. Multilingual and positive trends are discerned from the investigated HCPs. The findings inform linguistic researchers of the publication characteristics of the HCPs in the linguistics field and help them pinpoint the research trends and directions to exert their efforts in future studies.

## 1. Introduction

Citations, as a rule, exhibit a skewed distributional pattern over the academic publications: a few papers accumulate an overwhelming large citations while the majority are rarely, if ever, cited. Correspondingly, the highly cited papers (HCPs) receive the greatest amount of attention in the academia as citations are commonly regarded as a strong indicator of research excellence. For academic professionals, following HCPs is an efficient way to stay current with the developments in a field and to make better informed decisions regarding potential research topics and directions to exert their efforts. For academic institutions, government and private agencies, and generally the science policy makers, they keep a close eye on and take advantage of this visible indicator, citations, to make more informed decisions on research funding allocation and science policy formulation. Under the backdrop of ever-growing academic outputs, there is noticeable attention shift from publication quantity to publication quality. Many countries are developing research policies to identify “excellent” universities, research groups, and researchers (Danell, 2011). In a word, HCPs showcase high-quality research, encompass significant themes, and constitute a critical reference point in a research field as they are “gold bullion of science” (Smith, 2007).

## 2. Literature review

Bibliometrics, a term coined by Pritchard (1969), refers to the application of mathematical methods to the analysis of academic publications. Essentially this is a quantitative method to depict publication patterns within a given field based on a body of literature. There are many bibliometric studies on natural and social sciences in general (Hsu and Ho, 2014; Zhu and Lei, 2022) and on various specific disciplines such as management sciences (Liao et al., 2018), biomass research (Chen and Ho, 2015), computer sciences (Xie and Willett, 2013), and sport sciences (Mancebo et al., 2013; Ríos et al., 2013), etc. In these studies, researchers tracked developments, weighed research impacts, and highlighted emerging scientific fronts with bibliometric methods. In the field of linguistics, bibliometric studies all occurred in the past few years (van Doorslaer and Gambier, 2015; Lei and Liao, 2017; Gong et al., 2018; Lei and Liu, 2018, 2019). These bibliometric studies mostly examined a sub-area of linguistics, such as corpus linguistics (Liao and Lei, 2017), translation studies (van Doorslaer and Gambier, 2015), the teaching of Chinese as a second/foreign language (Gong et al., 2018), academic journals like *System* (Lei and Liu, 2018) or *Porta Linguarum* (Sabiote and Rodríguez, 2015), etc. Although Lei and Liu (2019) took the entire discipline of linguistics under investigation, their research is exclusively focused on *applied linguistics* and restricted in a limited number of journals (42 journals in total), leaving publications in other linguistics disciplines and qualified journals unexamined.

Over the recent years, a number of studies have been concerned with “excellent” papers or HCPs. For example, Small (2004) surveyed the HCPs authors’ opinions on why their papers are highly cited. The strong interest, the novelty, the utility, and the high importance of the work were among the most frequently mentioned. Most authors also considered that their selected HCPs are indeed based on their most important work in their academic career. Aksnes (2003) investigated the characteristics of HCPs and found that they were generally authored by a large number of scientists, often involving international collaboration. Some researchers even attempted to predict the HCPs by building mathematical models, implying “the first mover advantage in scientific publication” (Newman, 2008, 2014). In other words, papers published earlier in a field generally are more likely to accumulate more citations than those published later. Although many papers addressed HCPs from different perspectives, they held a common belief that HCPs are very different from less or zero cited papers and thus deserve utmost attention in academic research (Aksnes, 2003; Blessinger and Hrycaj, 2010; Yan et al., 2022).

Although an increased focus on research quality can be observed in different fields, opinions diverge on the range and the inclusion criterion of excellent papers. Are they ‘highly cited’, ‘top cited’, or ‘most frequently cited’ papers? Aksnes (2003) noted two different approaches to define a highly cited article, involving absolute or relative thresholds, respectively. An absolute threshold stipulates a minimum number of citations for identifying excellent papers while a relative threshold employs the percentile rank classes, for example, the top 10% most highly cited papers in a discipline or in a publication year or in a publication set. It is important to note that citations differ significantly in different fields and disciplines. A HCP in natural sciences generally accumulates more citations than its counterpart in social sciences. Thus, it is necessary to investigate HCPs from different fields separately or adopt different inclusion criterion to ensure a valid comparison.

The present study has been motivated by two considerations. First, the sizable number of publications of varied qualities in a scientific field makes it difficult or even impossible to conduct any reliable and effective literature research. Focusing on the quality publications, the HCPs in particular, might lend more credibility to the findings on trends. Second, HCPs can serve as a great platform to discover potentially important information for the development of a discipline and understand the past, present, and future of the scientific structure. Therefore, the present study aims to investigate the hot topics and publication trends in the Web of Science category of *linguistics or language & linguistics* (shortened as *linguistics* in later references) with bibliometric methods. The study aims to answer the following three questions:

1. Who are the most productive and impactful contributors of the HCPs in WoS category of *linguistics or language & linguistics* in terms of publication venues, authors, and countries?
2. What are the most frequently explored topics in HCPs?
3. What are the general research trends revealed from the HCPs?

## 3. Materials and methods

Different from previous studies which used an arbitrary inclusion threshold (e.g., Blessinger and Hrycaj, 2010; Hsu and Ho, 2014), we rely on Essential Science Indicator (ESI) to identify the HCPs. Developed by Clarivate, a leading company in the areas of bibliometrics and scientometrics, ESI reveals emerging science trends as well as influential individuals, institutions, papers, journals, and countries in any scientific fields of inquiry by drawing on the complete WoS databases. ESI has been chosen for the following three reasons. First, ESI adopts a stricter inclusion criterion for HCPs identification. That is, a paper is selected as a HCP only when its citations exceed the top 1% citation threshold in each of the 22 ESI subject categories. Second, ESI is widely used and recognized for its reliability and authority in identifying the top-charting work, generating “excellent” metrics including hot and highly cited papers. Third, ESI automatically updates its database to generate the most recent HCPs, especially suitable for trend studies for a specified timeframe.

### 3.1. Data source

The data retrieval was completed at the portal of our university library on June 20, 2022. The methods to retrieve the data are described in Table 1. The bibliometric indicators regarding the important contributors at journal/author/country levels were obtained. Specifically, after the research was completed, we clicked the “Analyze Results” bar on the result page for the detailed descriptive analysis of the retrieved bibliometric data.

**Table 1:** Retrieval strategies.

(from Clarivate Analytics Web of Science Core Collection)
Index: Social Science Citation Index (SSCI) and Arts & Humanities Citation Index (A&HCI)
Web of Science categories = linguistics or language & linguistics
Refined by: Highly Cited Papers

Several points should be noted about the search strategies. First, we searched the bibliometric data from two sub-databases of WoS core collection: Social Science Citation Index (SSCI) and Arts & Humanities Citation Index (A&HCI). There is no need to include the sub-database of Science Citation Index Expanded (SCI-EXPANDED) because publications in the linguistics field are almost exclusively indexed in SSCI and A&HCI journals. WoS core collection was chosen as the data source because it boasts one of the most comprehensive and authoritative databases of bibliometric information in the world. Many previous studies utilized WoS to retrieve bibliometric data. van Oorschot et al. (2018) and Ruggeri et al. (2019) even indicated that WoS meets the highest standards in terms of impact factor and citation counts and hence guarantees the validity of any bibliometric analysis. Second, we do not restrict the document types as HCPs selection informed by ESI only considers articles and reviews. Third, we do not set the date range as the dataset of ESI-HCPs is automatically updated regularly to include the most recent 10 years of publications.

The aforementioned query obtained a total of 143 HCPs published in 48 journals contributed by 352 authors of 226 institutions. We then downloaded the raw bibliometric parameters of the 143 HCPs for follow-up analysis including publication years, authors, publication titles, countries, affiliations, abstracts, citation reports, etc. A complete list of the 143 HCPs can be found in the Supplementary Material. We collected the most recent impact factor (IF) of each journal from the 2022 Journal Citation Reports (JCR).

### 3.2. Data analysis

#### 3.2.1. Citation analysis

**Figure 1:**
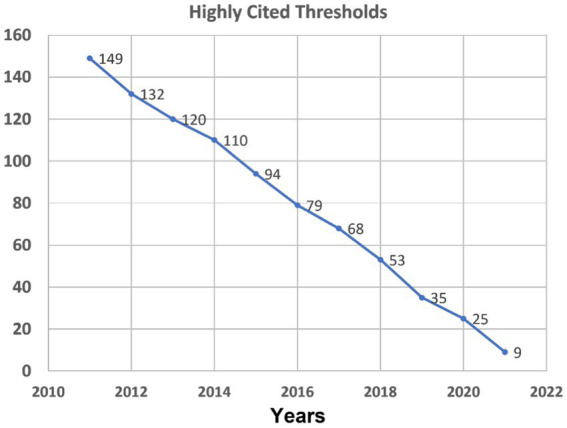
Highly cited thresholds in the research field of *Social Sciences, General.*

A citation threshold is the minimum number of citations obtained by ranking papers in a research field in descending order by citation counts and then selecting the top fraction or percentage of papers. In ESI, the highly cited threshold reveals the minimum number of citations received by the top 1% of papers from each of the 10 database years. In other words, a paper has to meet the minimum citation threshold that varies by research fields and by years to enter the HCP list. Of the 22 research fields in ESI, *Social Science, General* is a broad field covering a number of WoS categories including *linguistics* and *language & linguistics*. We checked the ESI official website to obtain the yearly highly cited thresholds in the research field of *Social Science*, *General* as shown in Figure 1 (https://esi.clarivate.com/ThresholdsAction.action). As we can see, the longer a paper has been published, the more citations it has to receive to meet the threshold. We then divided the raw citation numbers of HCPs with the Highly Cited Thresholds in the corresponding year to obtain the normalized citations for each HCP.

#### 3.2.2. Corpus analysis and manual checking

To determine the most frequently explored topics in these HCPs, we used both corpus-based analysis of word frequency and manual checking. Specifically, the more frequently a word or phrase occurs in a specifically designed corpus, the more likely it constitutes a research topic. In this study, we built an Abstract corpus with all the abstracts of the 143 HCPs, totaling 24,800 tokens. The procedures to retrieve the research topics in the Abstract corpus were as follows. First, the 143 pieces of abstracts were saved as separate .txt files in one folder. Second, AntConc (Anthony, 2022), a corpus analysis tool for concordancing and text analysis, was employed to extract lists of n-grams (2–4) in decreasing order of frequency. We also generated a list of individual nouns because sometimes individual nouns can also constitute research topics. Considering our small corpus data, we adopted both frequency (3) and range criteria (3) for topic candidacy. That is, a candidate n-gram must occur at least 3 times and in at least 3 different abstract files. The frequency threshold guarantees the importance of the candidate topics while the range threshold guarantees that the topics are not overly crowded in a few number of publications. In this process, we actually tested the frequency and range thresholds several rounds for the inclusion of all the potential topics. In total, we obtained 531 nouns, 1,330 2-grams, 331 3-grams, and 81 4-grams. Third, because most of the retrieved n-grams cannot function as meaningful research topics, we manually checked all the candidate items and discussed extensively to decide their roles as potential research topics until full agreements were reached. Finally, we read all the abstracts of the 143 HCPs to further validate their roles as research topics. In the end, we got 118 topic items in total.

## 4. Results

### 4.1. Main publication venues of HCPs

Of the 48 journals which published the 143 HCPs, 17 journals have contributed at least 3 HCPs (Table 2), around 71.33% of the total examined HCPs (102/143), indicating that HCPs tend to be highly concentrated in a limited number of journals. The three largest publication outlets of HCPs are *Bilingualism Language and Cognition* (16), *International Journal of Bilingual Education and Bilingualism* (11), and *Modern Language Journal* (10). Because each journal varies greatly in the number of papers published per year and the number of HCPs is associated with journal circulations, we divided the total number of papers (TP) in the examined years (2011–2021) with the number of the HCPs to acquire the HCP percentage for each journal (HCPs/TP). The three journals with the highest HCPs/TP percentage are *Annual Review of Applied Linguistics* (2.26), *Modern Language Journal* (2.08), and *Bilingualism Language and Cognition* (1.74), indicating that papers published in these journals have a higher probability to enter the HCPs list.

**Table 2:** Top 17 publication venues of HCPs.

Publication Titles	N	N%	TP	N/TP % (R)	TC	TC/HCP (R)	IF
*Bilingualism Language and Cognition*	16	11.19	918	1.74(3)	1,699	106.19(14)	4.763
*International Journal of Bilingual Education and Bilingualism*	11	7.70	829	1.33(6)	349	31.7(17)	3.165
*Modern Language Journal*	10	7.00	480	2.08(2)	1,353	135.3(12)	7.5
*Journal of Memory and Language*	7	4.90	730	0.96(10)	5,865	837.86(1)	4.521
*System*	7	4.90	1,472	0.48(15)	533	76.14(15)	4.518
*American Journal Of Speech Language Pathology*	6	4.20	1,040	0.58(13)	1,161	193.50(9)	4.018
*Applied Linguistics*	6	4.20	627	0.96(10)	1,186	197.67(8)	4.155
*Language Learning*	6	4.20	509	1.18(7)	975	162.50(11)	5.24
*Applied Linguistics Review*	4	2.80	281	1.42(5)	538	134.50(13)	3.063
*Computational Linguistics*	4	2.80	354	1.13(8)	2,135	533.75(2)	7.778
*Journal Of Pragmatics*	4	2.80	2,122	0.19(17)	1,215	303.75(3)	1.86
*Language Teaching*	4	2.80	371	1.08(9)	859	214.75(6)	4.769
*Language Teaching Research*	4	2.80	681	0.59(12)	213	53.25(16)	3.401
*Research On Language And Social Interaction*	4	2.80	244	1.64(4)	1,137	284.25(4)	4.158
*Annual Review of Applied Linguistics*	3	2.10	133	2.26(1)	755	251.67(5)	3.87
*Computer Assisted Language Learning*	3	2.10	588	0.51(14)	644	214.67(7)	5.964
*Language Cognition And Neuroscience*	3	2.10	813	0.37(16)	549	183.00(10)	2.842

N: the number of HCPs in each journal; N%: the percentage of HCPs in each journal in the total of 143 HCPs; TP: the total number of papers in the examined timespan (2011–2021); N/TP %: the percentage of HCPs in the total journal publications in the examined time span; TC/HCP: average citations of each HCP; R: journal ranking for the designated indicator; IF: Impact Factor in the year of 2022.

In terms of the general impact of the HCPs from each journal, we divided the number of HCPs with their total citations (TC) to obtain the average citations for each HCP (TC/HCP). The three journals with the highest TC/HCP are *Journal of Memory and Language* (837.86), *Computational Linguistics* (533.75), and *Journal of Pragmatics* (303.75). It indicates that even in the same WoS category, HCPs in different journals have strikingly different capability to accumulate citations. For example, the TC/HCP in *System* is as low as 31.73, which is even less than 4% of the highest TC/HCP in *Journal of Memory and Language*.

In regards to the latest journal impact factor (IF) in 2022, the top four journals with the highest IF are *Computational Linguistics (7.778)*, *Modern Language Journal* (7.5), *Computer Assisted Language Learning* (5.964), and *Language Learning* (5.24). According to the Journal Citation Reports (JCR) quantile rankings in WoS category of *linguistics*, all the journals on the list belong to the Q 1 (the top 25%), indicating that contributors are more likely to be attracted to contribute and cite papers in these prestigious high impact journals.

### 4.2. Authors of HCPs

A total of 352 authors had their names listed in the 143 HCPs, of whom 33 authors appeared in at least 2 HCPs as shown in Table 3. We also provided in Table 3 other indicators to evaluate the authors’ productivity and impact including the total number of citations (TC), the number of citations per HCP, and the number of First author or Corresponding author HCPs (FA/CA). The reason we include the FA/CA indicator is that first authors and corresponding authors are usually considered to contribute the most and should receive greater proportion of credit in academic publications (Marui et al., 2004; Dance, 2012).

**Table 3:** Authors with at least 2 HCPs.

Author	Affiliations	N	FA/CA	TC	C/HCP
*Dewaele JM*	Birkbeck Univ London	7	2	492	70.3
*Li CC*	Huazhong Univ Sci & Technol	5	5	215	43
*Saito K*	UCL	5	2	576	115.2
*Garcia O*	CUNY	3	1	543	181
*Macintyre PD*	Cape Breton Univ	3	2	292	97.33
*Mondada L*	Univ Basel	3	3	392	130.7
*Norton B*	Univ British Columbia	3	1	915	305
*Otheguy R*	CUNY	3	2	543	181
*Plonsky L*	No Arizona Univ	3	1	676	225.3
*Larsen-freeman D*	Univ Michigan	2	1	375	187.5
*Zhang LJ*	Univ Auckland	2	0	98	49
*Wei L*	UCL	2	2	956	478
*Luk G*	York Univ	2	2	241	120.5
*Mercer S*	Karl Franzens Univ Graz	2	0	204	102
*Ortega L*	Georgetown Univ	2	1	395	197.5
*Vasishth S*	Univ Potsdam	2	0	694	347
*Baayen RH*	Univ Tubingen	2	1	280	140
*Brysbaert M*	Univ Ghent	2	1	162	81
*Canagarajah S*	Penn State Univ	2	2	537	268.5
*Derakhshan A*	Golestan Univ	2	1	77	38.5
*Dornyei Z*	Univ Nottingham	2	1	281	140.5
*Gao XS*	Univ New South Wales	2	1	86	43
*Gong Y*	Ningbo Univ	2	2	61	30.5
*Gregersen T*	Amer Univ Sharjah	2	0	204	102
*Jiang GY*	Xiamen Univ	2	2	127	63.5
*Kliegl R*	Univ Potsdam	2	0	694	347
*Li P*	Hong Kong Polytech Univ	2	2	148	74
*Pennycook A*	Univ Technol Sydney	2	2	206	103
*Piller I*	Macquarie University	2	2	226	113
*Polinsky M*	Univ Maryland	2	1	292	146
*Otheguy R*	CUNY	2	2	475	237.5
*Rothman J*	UiT Arctic Univ Norway;	2	1	146	73
*Al-hoorie AH*	Univ Nottingham	2	0	124	62

N: number of HCPs from each author; FA/CA: first author or corresponding author HCPs; TC: total citations of the HCPs from each author; C/HCP: average citations per HCP for each author.

In terms of the number of HCPs, *Dewaele JM* from *Birkbeck Univ London* tops the list with 7 HCPs with total citations of 492 (TC = 492), followed by *Li C* from *Huazhong Univ Sci & Technol* (#HCPs = 5; TC = 215) and *Saito K from UCL* (#HCPs = 5; TC = 576). It is to be noted that both *Li C* and *Saito K* have close academic collaborations with *Dewaele JM*. For example, 3 of the 5 HCPs by *Li C* are co-authored with *Dewaele JM*. The topics in their co-authored HCPs are mostly about foreign language learning emotions such as *boredom*, *anxiety*, *enjoyment*, *the measurement*, and *positive psychology*.

In regards to TC, *Li, W*. from *UCL* stands out as the most influential scholar among all the listed authors with total citations of 956 from 2 HCPs, followed by *Norton B* from *Univ British Columbia* (TC = 915) and *Vasishth S* from *Univ Potsdam* (TC = 694). The average citations per HCP from them are also the highest among the listed authors (478, 305, 347, respectively). It is important to note that *Li, W.’*s 2 HCPs are his groundbreaking works on *translanguaging* which almost become must-reads for anyone who engages in translanguaging research (Li, 2011, 2018). Besides, *Li, W.* single authors his 2 HCPs, which is extremely rare as HCPs are often the results from multiple researchers. *Norton B*’s HCPs are exploring some core issues in applied linguistics such as *identity* and *investment*, *language learning*, and *social change* that are considered the foundational work in its field (Norton and Toohey, 2011; Darvin and Norton, 2015).

From the perspective of FA/CA papers, *Li C* from *Huazhong Univ Sci and Technol* is prominent because she is the first author of all her 5 HCPs. Her research on *language learning emotions* in the Chinese context is gaining widespread recognition (Li et al., 2018, 2019, 2021; Li, 2019, 2021). However, as a newly emerging researcher, most of her HCPs are published in the very recent years and hence accumulate relatively fewer citations (TC = 215). *Mondada L* from *Univ Basel* follows closely and single authors her 3 HCPs. Her work is mostly devoted to *conversation analysis*, *multimodality*, and *social interaction* (Mondada, 2016, 2018, 2019).

We need to mention the following points regarding the productive authors of HCPs. First, when we calculated the number of HCPs from each author, only the papers published in the journals indexed in the investigated WoS categories were taken in account (*linguistics; language & linguistics*), which came as a compromise to protect the linguistics oriented nature of the HCPs. For example, *Brysbaert M* from *Ghent University* claimed a total of 8 HCPs at the time of the data retrieval, of which 6 HCPs were published in WoS category of *psychology* and more psychologically oriented, hence not included in our study. Besides, all the authors on the author list were treated equally when we calculated the number of HCPs, disregarding the author ordering. That implies that some influential authors may not be able to enter the list as their publications are comparatively fewer. Second, as some authors reported different affiliations at their different career stages, we only provide their most recent affiliation for convenience. Third, it is highly competitive to have one’s work selected as HCPs. The fact that a majority of the HCPs authors do not appear in our productive author list does not diminish their great contributions to this field. The rankings in Table 3 does not necessarily reflect the recognition authors have earned in academia at large.

### 4.3. Productive countries of HCPs

In total, the 143 HCPs originated from 33 countries. The most productive countries that contributed at least three HCPs are listed in Table 4. The *USA* took an overwhelming lead with 59 HCPs, followed distantly by *England* with 31 HCPs. They also boasted the highest total citations (TC = 15,770; TC = 9,840), manifesting their high productivity and strong influence as traditional powerhouses in linguistics research. In regards to the average citations per HCP, *Germany*, *England* and the *USA* were the top three countries (TC/HCP = 281.67, 281.14, and 267.29, respectively). Although China held the third position with 19 HCPs published, its TC/HCP is the third from the bottom (TC/HCP = 66.84). One of the important reasons is that 13 out of the 19 HCPs contributed by scholars in China are published in the year of 2020 or 2021. The newly published HCPs may need more time to accumulate citations. Besides, 18 out of the 19 HCPs in China are first author and/or corresponding authors, indicating that scholars in China are becoming more independent and gaining more voice in English linguistics research.

**Table 4:** Top 18 countries with at least 3 HCPs.

Countries	HCPs	HCPs %	TC	C/HCP	FA/CA
*USA*	59	41.26	15,770	267.29	53
*England*	35	24.48	9,840	281.14	26
*China*	19	13.29	1,270	66.84	18
*Canada*	15	10.49	3,981	265.40	13
*Australia*	12	8.39	1,061	88.42	10
*Germany*	9	6.29	2,535	281.67	5
*Netherlands*	6	4.20	469	78.17	5
*Iran*	5	3.50	216	43.20	5
*Austria*	4	2.80	668	167.00	1
*Japan*	4	2.80	540	135.00	0
*Norway*	4	2.80	549	137.25	2
*Spain*	4	2.80	539	134.75	3
*Belgium*	3	2.10	274	91.33	3
*Finland*	3	2.10	521	173.67	3
*Israel*	3	2.10	523	174.33	0
*New Zealand*	3	2.10	115	38.33	1
*Switzerland*	3	2.10	393	131.00	3
*U Arab Emirates*	3	2.10	232	77.33	1

Two points should be noted here as to the productive countries. First, we calculated the HCP contributions from the country level instead of the region level. In other words, HCP contributions from different regions of the same country will be combined in the calculation. For example, HCPs from *Scotland* were added to the HCPs from *England*. HCPs from *Hong Kong*, *Macau*, and *Taiwan* are put together with the HCPs from *Mainland China*. In this way, a clear picture of the HCPs on the country level can be painted. Second, we manually checked the address information of the first author and corresponding author for each HCP. There are some cases where the first author or the corresponding author may report affiliations from more than one country. In this case, every country in their address list will be treated equally in the FA/CA calculation. In other word, a HCP may be classified into more than one country because of the different country backgrounds of the first and/or the corresponding author.

### 4.4. Top 20 HCPs

The top 20 HCPs with the highest normed citations are listed in decreasing order in Table 5. The top cited publications can guide us to better understand the development and research topics in recent years.

**Table 5:** Top 20 HCPs.

#	RC	NC	Authors	Title (Publication Year)	Journals
1	4,677	38.88	Barr, D.J., et al.	Random effects structure for confirmatory hypothesis testing: keep it maximal (2013)	Journal of Memory and Language
2	519	20.24	Lee, JB & Azios, JH	Facilitator Behaviors Leading to Engagement and Disengagement in Aphasia Conversation Groups (2020)	American Journal of Speech-Language Pathology
3	583	8.57	Matuschek, H, et al.	Balancing type I error and power in linear mixed models (2017)	Journal of Memory and Language
4	1,313	8.42	Taboada, M, et al.	Lexicon-Based methods for sentiment analysis (2011)	Computational Linguistics
5	374	7.06	Li, W	Translanguaging as a Practical Theory of language (2018)	Applied Linguistics
6	136	5.44	Alva Manchego, F, et al.	Data-Driven sentence simplification: survey and benchmark (2020)	Computational Linguistics
7	693	5.22	Heritage, J	The epistemic engine: sequence organization and territories of language (2012)	Research on Language and Social Interaction
8	46	5.11	Zhang, Q; Yang, T	Reflections on the medium of instruction for ethnic minorities in Xinjiang: the case of bilingual schools in Urumqi (2021)	International Journal of Bilingual Education and Bilingualism
9	560	5.08	Plonsky, L; Oswald, FL	How big is big? interpreting effect sizes in L2 research (2014)	Language Learning
10	371	4.65	Kuperberg, GR; Jaeger, TF	What do we mean by prediction in language comprehension? (2016)	Language Cognition and Neuroscience
11	41	4.56	Greenier, V, et al.	Emotion regulation and psychological well-being in teacher work engagement: a case of British and Iranian English…(2021)	System
12	240	4.49	Macaro, E, et al.	A systematic review of English medium instruction in higher education (2018)	Language Teaching
13	406	4.26	Otheguy, R, et al.	Clarifying translanguaging and deconstructing named languages:a perspective from linguistics (2015)	Applied Linguistics Review
14	107	4.24	Schad, DJ, et al.	How to capitalize on *a priori* contrasts in linear(mixed) models: a tutorial (2020)	Journal of Memory and Language
15	38	4.22	Shirvan, ME; Taherian, T	Longitudinal examination of university students’ foreign language enjoyment and foreign language classroom anxiety…(2021)	International Journal of Bilingual Education and Bilingualism
16	101	4.04	MacIntyre, PD, et al.	Language teachers’ coping strategies during the Covid-19 conversion to online…(2020)	System
17	320	4.03	Atkinson, D, et al.	A transdisciplinary framework for SLA in a multilingual world (2016)	Modern Language Journal
18	36	4.00	Jin, YX; Zhang, LJ	The dimensions of foreign language classroom enjoyment and their effect on foreign language achievement (2021)	International Journal of Bilingual Education and Bilingualism
19	35	3.89	Derakhshan, A, et al.	Boredom in online classes in the Iranian EFL contexts: sources and solutions (2021)	System
20	575	3.83	Wei, L	Moment analysis and translanguaging space: discursive construction of identities…(2011)	Journal of Pragmatics

To save space, not full information about the HCPs is given. Some article titles have been abbreviated if they are too lengthy; for the authors, we report the first two authors and use “et al” if there are three authors or more; RC: raw citations; NC: normalized citations

By reading the titles and the abstracts of these top HCPs, we categorized the topics of the 20 HCPs into the following five groups: (i) *statistical and analytical methods* in (psycho)linguistics such as sentimental analysis, sentence simplification techniques, effect sizes, linear mixed models (#1, 3, 4, 6, 9, 14), (ii) *language learning/teaching emotions* such enjoyment, anxiety, boredom, stress (#11, 15, 16, 18, 19), (iii) *translanguaging or multilinguilism* (#5, 13, 20, 17), (iv) *language perception* (#2, 7, 10), (v) *medium of instruction* (#8, 12). It is no surprise that 6 out of the top 20 HCPs are about statistical methods in linguistics because language researchers aspire to employ statistics to make their research more scientific. Besides, we noticed that the papers on language teaching/learning emotions on the list are all published in the year of 2020 and 2021, indicating that these emerging topics may deserve more attention in future research. We also noticed two Covid-19 related articles (#16, 19) explored the emotions teachers and students experience during the pandemic, a timely response to the urgent need of the language learning and teaching community.

It is of special interest to note that papers from the journals indexed in multiple JCR categories seem to accumulate more citations. For example, *Journal of Memory and Language*, *American Journal of Speech-Language Pathology*, and *Computational Linguistics* are indexed both in SSCI and SCIE and contribute the top 4 HCPs, manifesting the advantage of these hybrid journals in amassing citations compared to the conventional language journals. Besides, different to findings from Yan et al. (2022) that most of the top HCPs in the field of *radiology* are reviews in document types, 19 out of the top 20 HCPs are research articles instead of reviews except Macaro et al. (2018).

### 4.5. Most frequently explored topics of HCPs

After obtaining the corpus based topic items, we read all the titles and abstracts of the 143 HCPs to further validate their roles as research topics. Table 6 presents the top research topics with the observed frequency of 5 or above. We grouped these topics into five broad categories: *bilingual-related, language learning/teaching-related, psycho/pathological/cognitive linguistics-related, methods and tools-related, and others*. The observed frequency count for each topic in the abstract corpus were included in the brackets. We found that about 34 of the 143 HCPs are exploring bilingual related issues, the largest share among all the categorized topics, testifying its academic popularity in the examined timespan. Besides, 30 of the 143 HCPs are investigating language learning/teaching-related issues, with topics ranging from learners (e.g., EFL learners, individual difference) to multiple learning variables (e.g., learning strategy, motivation, agency). The findings here will be validated by the analysis of the keywords.

**Table 6:** Categorization of the most explored research topics.

Categories	N	hot topic items
Multilingual-related	34	Multilingualism(127), translanguaging(42), heritage language/speakers/learners(31), language/education policy(6)
Language learning/teaching-related	30	Language/writing development(35), academic writing/vocabulary/publishing(22), learning strategy(20), motivation(17), individual differences(13), CLIL(11), agency(11), flipped classroom(9), self-efficacy(9), EFL learner(7), ELF (7), early language(7)
Psycho/pathological/cognitive linguistics-related	25	Emotion(47), FLE(42), cognition(39), anxiety(35), FLCA(30), stuttering(21), anxiety/language/fluency disorder(16), boredom(14), language impairment(14), brain(11), working memory(9), speech language pathology/therapy/pathologists(7), positive psychology(6), language ideology(5)
Methods and tools-related	16	Model(67), review (35), qualitative data(14), quantitative data(8), corpus-based studies/teaching(6), longitudinal study/analysis(5), sentiment analysis(5), meta-analysis(5), eye tracking(4), mixed method(4)
Others	38	Lexical(25), identity(21), social interaction/difficulties(17), sematic models/mapping(15), Covid-19(9)

N: the number of the HCPs in each topic category; ELF: English as a lingua franca; CLIL: content and language integrated learning; FLE: foreign language enjoyment; FLCA: foreign language classroom anxiety

Several points should be mentioned regarding the topic candidacy. First, for similar topic expressions, we used a cover term and added the frequency counts. For example, *multilingualism* is a cover term for *bilinguals, bilingualism, plurilingualism, and multilingualism*. Second, for nouns of singular and plural forms (e.g., *emotion* and *emotions*) or for items with different spellings (e.g., *meta analysis* and *meta analyses*), we combined the frequency counts. Third, we found that some longer items (3 grams and 4 grams) could be subsumed to short ones (2 grams or monogram) without loss of essential meaning (e.g., *working memory* from *working memory capacity*). In this case, the shorter ones were kept for their higher frequency. Fourth, some highly frequent terms were discarded because they were too general to be valuable topics in language research, for example, *applied linguistics*, *language use*, *second language*.

## 5. Discussion and implications

Based on 143 highly cited papers collected from the WoS categories of *linguistics*, the present study attempts to present a bird’s eye view of the publication landscape and the most updated research themes reflected from the HCPs in the linguistics field. Specifically, we investigated the important contributors of HCPs in terms of journals, authors and countries. Besides, we spotlighted the research topics by corpus-based analysis of the abstracts and a detailed analysis of the top HCPs. The study has produced several findings that bear important implications.

The first finding is that the HCPs are highly concentrated in a limited journals and countries. In regards to journals, those in the spheres of bilingualism and applied linguistics (e.g., language teaching and learning) are likely to accumulate more citations and hence to produce more HCPs. Journals that focus on bilingualism from a linguistic, psycholinguistic, and neuroscientific perspective are the most frequent outlets of HCPs as evidenced by the top two productive journals of HCPs, *Bilingualism Language and Cognition* and *International Journal of Bilingual Education and Bilingualism*. This can be explained by the multidisciplinary nature of bilingual-related research and the development of cognitive measurement techniques. The merits of analyzing publication venues of HCPs are two folds. One the one hand, it can point out which sources of high-quality publications in this field can be inquired for readers as most of the significant and cutting-edge achievements are concentrated in these prestigious journals. On the other hand, it also provides essential guidance or channels for authors or contributors to submit their works for higher visibility.

In terms of country distributions, the traditional powerhouses in linguistics research such as the *USA* and *England* are undoubtedly leading the HCP publications in both the number and the citations of the HCPs. However, developing countries are also becoming increasing prominent such as *China* and *Iran*, which could be traceable in the funding and support of national language policies and development policies as reported in recent studies (Ping et al., 2009; Lei and Liu, 2019). Take China as an example. Along with economic development, China has given more impetus to academic outputs with increased investment in scientific research (Lei and Liao, 2017). Therefore, researchers in China are highly motivated to publish papers in high-quality journals to win recognition in international academia and to deal with the publish or perish pressure (Lee, 2014). These factors may explain the rise of China as a new emerging research powerhouse in both natural and social sciences, including English linguistics research.

The second finding is the multilingual trend in linguistics research. The dominant clustering of topics regarding multilingualism can be understood as a timely response to the multilingual research fever (May, 2014). 34 out of the 143 HCPs have such words as *bilingualism, bilingual, multilingualism*, *translanguaging*, etc., in their titles, reflecting a strong multilingual tendency of the HCPs. Multilingual-related HCPs mainly involve three aspects: multilingualism from the perspectives of psycholinguistics and cognition (e.g., Luk et al., 2011; Leivada et al., 2020); multilingual teaching (e.g., Schissel et al., 2018; Ortega, 2019; Archila et al., 2021); language policies related to multilingualism (e.g., Shen and Gao, 2018). As a pedagogical process initially used to describe the bilingual classroom practice and also a frequently explored topic in HCPs, *translanguaging* is developed into an applied linguistics theory since Li’s *Translanguaging as a Practical Theory of Language* (Li, 2018). The most common collocates of *translanguaging* in the Abstract corpus are *pedagogy/pedagogies, practices, space/spaces*. There are two main reasons for this multilingual turn. First, the rapid development of globalization, immigration, and overseas study programs greatly stimulate the use and research of multiple languages in different linguistic contexts. Second, in many non-English countries, courses are delivered through languages (mostly English) besides their mother tongue (Clark, 2017). Students are required to use multiple languages as resources to learn and understand subjects and ideas. The burgeoning body of English Medium Instruction literature in higher education is in line with the rising interest in multilingualism. Due to the innate multidisciplinary nature, it is to be expected that, multilingualism, the topic du jour, is bound to attract more attention in the future.

The third finding is the application of Positive Psychology (PP) in second language acquisition (SLA), that is, the positive trend in linguistic research. In our analysis, 20 out of 143 HCPs have words or phrases such as *emotions, enjoyment, boredom, anxiety*, and *positive psychology* in their titles, which might signal a shift of interest in the psychology of language learners and teachers in different linguistic environments. Our study shows Foreign language enjoyment (FLE) is the most frequently explored emotion, followed by foreign language classroom anxiety (FLCA), the learners’ metaphorical left and right feet on their journey to acquiring the foreign language (Dewaele and MacIntyre, 2016). In fact, the topics of PP are not entirely new to SLA. For example, studies of language motivations, affections, and good language learners all provide roots for the emergence of PP in SLA (Naiman, 1978; Gardner, 2010). In recent years, both research and teaching applications of PP in SLA are building rapidly, with a diversity of topics already being explored such as positive education and PP interventions. It is to be noted that SLA also feeds back on PP theories and concepts besides drawing inspirations from it, which makes it “an area rich for interdisciplinary cross-fertilization of ideas” (Macintyre et al., 2019).

It should be noted that subjectivity is involved when we decide and categorize the candidate topic items based on the Abstract corpus. However, the frequency and range criteria guarantee that these items are actually more explored in multiple HCPs, thus indicating topic values for further investigation. Some high frequent n-grams are abandoned because they are too general or not meaningful topics. For example, *applied linguistics* is too broad to be included as most of the HCPs concern issues in this research line instead of theoretical linguistics. By meaningful topics, we mean that the topics can help journal editors and readers quickly locate their interested fields (Lei and Liu, 2019), as the author keywords such as *bilingualism*, *emotions*, and *individual differences*. The examination of the few 3/4-grams and monograms (mostly nouns) revealed that most of them were either not meaningful topics or they could be subsumed in the 2-grams. Besides, there is inevitably some overlapping in the topic categorizations. For example, some topics in the language teaching and learning category are situated and discussed within the context of multilingualism. The merits of topic categorizations are two folds: to better monitor the overlapping between the Abstract corpus-based topic items and the keywords; to roughly delineate the research strands in the HCPs for future research.

It should also be noted that all the results were based on the retrieved HCPs only. The study did not aim to paint a comprehensive and full picture of the whole landscape of linguistic research. Rather, it specifically focused on the most popular literature in a specified timeframe, thus generating the snapshots or trends in linguistic research. One of the important merits of this methodology is that some newly emerging but highly cited researchers can be spotlighted and gain more academic attention because only the metrics of HCPs are considered in calculation. On the contrary, the exclusion of some other highly cited researchers in general such as Rod Ellis and Ken Hyland just indicates that their highly cited publications are not within our investigated timeframe and cannot be interpreted as their diminishing academic influence in the field. Besides, the study does not consider the issue of collaborators or collaborations in calculating the number of HCPs for two reasons. First, although some researchers are regular collaborators such as Li CC and Dewaele JM, their individual contribution can never be undermined. Second, the study also provides additional information about the number of the FA/CA HCPs from each listed author, which may aid readers in locating their interested research.

We acknowledge that our study has some limitations that should be addressed in future research. First, our study focuses on the HCPs extracted from WoS SSCI and A&HCI journals, the alleged most celebrated papers in this field. Future studies may consider including data from other databases such as Scopus to verify the findings of the present study. Second, our Abstract corpus-based method for topic extraction involved human judgement. Although the final list was the result of several rounds of discussions among the authors, it is difficult or even impossible to avoid subjectivity and some worthy topics may be unconsciously missed. Therefore, future research may consider employing automatic algorithms to extract topics. For example, a dependency-based machine learning approach can be used to identify research topics (Zhu and Lei, 2021).

## Data availability statement

The datasets presented in this study can be found in online repositories. The names of the repository/repositories and accession number(s) can be found in the article/supplementary material.

## Author contributions

SY: conceptualization and methodology. SY and LZ: writing-review and editing and writing-original draft. All authors contributed to the article and approved the submitted version.

## Funding

This work was supported by Humanities and Social Sciences Youth Fund of China MOE under the grant 20YJC740076 and 18YJC740141.

## Conflict of interest

The authors declare that the research was conducted in the absence of any commercial or financial relationships that could be construed as a potential conflict of interest.

## Publisher’s note

All claims expressed in this article are solely those of the authors and do not necessarily represent those of their affiliated organizations, or those of the publisher, the editors and the reviewers. Any product that may be evaluated in this article, or claim that may be made by its manufacturer, is not guaranteed or endorsed by the publisher.

## Supplementary material

The Supplementary material for this article can be found online at: https://www.frontiersin.org/articles/10.3389/fpsyg.2022.1052586/full#supplementary-material

## References

[ref1] Aksnes D. W. (2003). Characteristics of highly cited papers. Res. Eval. 12, 159–170. doi: 10.3152/147154403781776645

[ref2] Anthony L. (2022). AntConc (version 4.0.5) Tokyo, Japan: Waseda University. Available at: https://www.laurenceanthony.net/software (Accessed June 20, 2022).

[ref3] Archila P. A. Molina J. Truscott de Mejía A.-M. (2021). Fostering bilingual scientific writing through a systematic and purposeful code-switching pedagogical strategy. Int. J. Biling. Educ. Biling. 24, 785–803. doi: 10.1080/13670050.2018.1516189

[ref4] Blessinger K. Hrycaj P. (2010). Highly cited articles in library and information science: an analysis of content and authorship trends. Libr. Inf. Sci. Res. 32, 156–162. doi: 10.1016/j.lisr.2009.12.007

[ref5] Chen H. Ho Y. S. (2015). Highly cited articles in biomass research: a bibliometric analysis. Renew. Sust. Energ. Rev. 49, 12–20. doi: 10.1016/j.rser.2015.04.060

[ref6] Clark S. (2017). Translanguaging in higher education: beyond monolingual ideologies. Int. J. Biling. Educ. Biling. 22, 1048–1051. doi: 10.1080/13670050.2017.1322568

[ref7] Dance A. (2012). Authorship: Who’s on first? Nature 489, 591–593. doi: 10.1038/nj7417-591a, PMID: 23025001

[ref8] Danell R. (2011). Can the quality of scientific work be predicted using information on the author’s track record? J. Am. Soc. Inf. Sci. Technol. 62, 50–60. doi: 10.1002/asi.21454

[ref9] Darvin R. Norton B. (2015). Identity and a model of Investment in Applied Linguistics. Annu. Rev. Appl. Linguist. 35, 36–56. doi: 10.1017/S0267190514000191

[ref10] Dewaele J.-M. MacIntyre P. D. (2016). “Foreign language enjoyment and foreign language classroom anxiety: the right and left feet of the language learner” in Positive psychology in SLA. eds. Peter D. M. Tammy G. Sarah M. (Bristol, Blue Ridge Summit: Multilingual Matters), 215–236.

[ref11] Gardner R. (2010). Motivation and second language acquisition: The socio-educational model. New York: Peter Lang.

[ref12] Gong Y. Lyu B. Gao X. (2018). Research on teaching Chinese as a second or foreign language in and outside mainland China: a bibliometric analysis. Asia Pac. Educ. Res. 27, 277–289. doi: 10.1007/s40299-018-0385-2

[ref13] Hsu Y. Ho Y. S. (2014). Highly cited articles in health care sciences and services field in science citation index Expanded. Methods Inf. Med. 53, 446–458. doi: 10.3414/ME14-01-0022, PMID: 25301516

[ref14] Lee I. (2014). Publish or perish: the myth and reality of academic publishing. Lang. Teach. 47, 250–261. doi: 10.1017/S0261444811000504

[ref15] Lei L. Liao S. (2017). Publications in linguistics journals from mainland China, Hong Kong, Taiwan, and Macau (2003–2012): a bibliometric analysis. J. Quant. Ling. 24, 54–64. doi: 10.1080/09296174.2016.1260274

[ref16] Lei L. Liu D. (2018). The research trends and contributions of System’s publications over the past four decades (1973–2017): a bibliometric analysis. System 80, 1–13. doi: 10.1016/j.system.2018.10.003

[ref17] Lei L. Liu D. (2019). Research trends in applied linguistics from 2005 to 2016: a bibliometric analysis and its implications. Appl. Linguis. 40, 540–561. doi: 10.1093/applin/amy003

[ref18] Leivada E. Westergaard M. Duabeitia J. A. Rothman J. (2020). On the phantom-like appearance of bilingualism effects on neurocognition: (how) should we proceed? Biling. Lang. Congn. 24, 197–210. doi: 10.1017/S1366728920000358

[ref19] Li W. (2011). Moment analysis and translanguaging space: discursive construction of identities by multilingual Chinese youth in Britain. Energy Fuel 43, 1222–1235. doi: 10.1016/j.pragma.2010.07.035

[ref20] Li W. (2018). Translanguaging as a practical theory of language. Appl. Linguis. 39, 9–30. doi: 10.1093/applin/amx039

[ref21] Li C. (2019). A positive psychology perspective on Chinese EFL students’ trait emotional intelligence, foreign language enjoyment and EFL learning achievement. J. Multiling. Multicult. Dev. 41, 246–263. doi: 10.1080/01434632.2019.1614187

[ref22] Li C. (2021). A control-value theory approach to boredom in English classes among university students in China. Mod. Lang. J. 105, 317–334. doi: 10.1111/modl.12693

[ref23] Li C. Dewaele J. M. Hu Y. (2021). Foreign language learning boredom: conceptualization and measurement. Appl. Ling. Rev. doi: 10.1515/applirev-2020-0124

[ref24] Li C. Dewaele J. M. Jiang G. (2019). The complex relationship between classroom emotions and EFL achievement in China. Appl. Ling. Rev. 11, 485–510. doi: 10.1515/applirev-2018-0043

[ref25] Li C. Jiang G. Jean-Marc D. (2018). Understanding Chinese high school students’ foreign language enjoyment: validation of the Chinese version of the foreign language enjoyment scale. System 76, 183–196. doi: 10.1016/j.system.2018.06.004

[ref26] Liao S. Lei L. (2017). What we talk about when we talk about corpus: a bibliometric analysis of corpus-related research in linguistics (2000-2015). Glottometrics 38, 1–20.

[ref27] Liao H. Tang M. Li Z. Lev B. (2018). Bibliometric analysis for highly cited papers in operations research and management science from 2008 to 2017 based on essential science indicators. Omega 88, 223–236. doi: 10.1016/j.omega.2018.11.005

[ref28] Luk G. Sa E. D. Bialystok E. (2011). Is there a relation between onset age of bilingualism and enhancement of cognitive control? Biling. Lang. Cogn. 14, 588–595. doi: 10.1017/S1366728911000010

[ref01] Macaro E. Curle S. Pun J. Dearden J. (2018). A systematic review of English medium instruction in higher education. Lang. Teach. 51, 36–76. doi: 10.1017/S0261444817000350

[ref29] Macintyre P. Gregersen T. Mercer S. (2019). Setting an agenda for positive psychology in SLA: theory, practice, and research. Mod. Lang. J. 103, 262–274. doi: 10.1111/modl.12544

[ref30] Mancebo F. P. Sapena A. F. Herrera M. V. González L. Toca H. Benavent R. A. (2013). Scientific literature analysis of judo in web of science. Arch. Budo 9, 81–91. doi: 10.12659/AOB.883883

[ref31] Marui M. Bozikov J. Katavi V. Hren D. Kljakovi-Gapi M. Marui A. (2004). Authorship in a small medical journal: a study of contributorship statements by corresponding authors. Sci. Eng. Ethics 10, 493–502. doi: 10.1007/s11948-004-0007-7, PMID: 15362706

[ref32] May S. (2014). The multilingual turn: Implications for SLA, TESOL and bilingual education. New York: Routledge.

[ref33] Mondada L. (2016). Challenges of multimodality: language and the body in social interaction. J. Socioling. 20, 336–366. doi: 10.1111/josl.1_12177

[ref34] Mondada L. (2018). Multiple temporalities of language and body in interaction: challenges for transcribing multimodality. Res. Lang. Soc. Interact. 51, 85–106. doi: 10.1080/08351813.2018.1413878

[ref35] Mondada L. (2019). Contemporary issues in conversation analysis: embodiment and materiality, multimodality and multisensoriality in social interaction. J. Pragmat. 145, 47–62. doi: 10.1016/j.pragma.2019.01.016

[ref36] Naiman N. (1978). The good language learner. Clevedon, UK: Multilingual Matters.

[ref37] Newman M. (2008). The first-mover advantage in scientific publication. Eplasty 86, 68001–68006. doi: 10.1209/0295-5075/86/68001

[ref38] Newman M. (2014). Prediction of highly cited papers. Eplasty 105:28002. doi: 10.1209/0295-5075/105/28002

[ref39] Norton B. Toohey K. (2011). Identity, language learning, and social change. Lang. Teach. 44, 412–446. doi: 10.1017/S0261444811000309

[ref40] Ortega L. (2019). SLA and the study of equitable multilingualism. Mod. Lang. J. 103, 23–38. doi: 10.1111/modl.12525

[ref41] Ping Z. Thijs B. Glnzel W. (2009). Is China also becoming a giant in social sciences? Scientometrics 79, 593–621. doi: 10.1007/s11192-007-2068-x

[ref42] Pritchard A. (1969). Statistical bibliography or bibliometrics. J. Doc. 25, 348–349.

[ref43] Ríos L. J. C. Tamao I. M. Olmos J. (2013). Bibliometric study (1922-2009) on rugby articles in research journals. South Afr. J. Res. Sport Phys. Educ. Rec. 17, 313–109. doi: 10.3176/tr.2013.3.06

[ref44] Ruggeri G. Orsi L. Corsi S. (2019). A bibliometric analysis of the scientific literature on Fairtrade labelling. Int. IJC 43, 134–152. doi: 10.1111/ijcs.12492

[ref45] Sabiote C. R. Rodríguez J. A. (2015). Bibliometric study and methodological quality indicators of the journal porta Linguarum during six year period 2008-2013. Porta Ling. 24, 135–150. doi: 10.30827/Digibug.53866

[ref46] Schissel J. L. De Korne H. López-Gopar M. E. (2018). Grappling with translanguaging for teaching and assessment in culturally and linguistically diverse contexts: teacher perspectives from Oaxaca, Mexico. Int. J. Biling. Educ. Biling. 24, 340–356. doi: 10.1080/13670050.2018.1463965

[ref47] Shen Q. Gao X. (2018). Multilingualism and policy making in greater China: ideological and implementational spaces. Lang. Policy 18, 1–16. doi: 10.1007/s10993-018-9473-7

[ref48] Small H. (2004). Why authors think their papers are highly cited. Scientometrics 60, 305–316. doi: 10.1023/B:SCIE.0000034376.55800.18

[ref49] Smith D. R. (2007). The New Zealand timber economy, 1840–1935. N. Z. Med. J. 120, U2871–U2313. doi: 10.1016/0305-7488(90)90044-C, PMID: 18157197

[ref50] van Doorslaer L. Gambier Y. (2015). Measuring relationships in translation studies. On affiliations and keyword frequencies in the translation studies bibliography. Perspectives 23, 305–319. doi: 10.1080/0907676X.2015.1026360

[ref51] van Oorschot J. A. W. H. Hofman E. Halman J. (2018). A bibliometric review of the innovation adoption literature. Technol. Forecast. Soc. Chang. 134, 1–21. doi: 10.1016/j.techfore.2018.04.032

[ref52] Xie Z. Willett P. (2013). The development of computer science research in the People’s republic of China 2000–2009: a bibliometric study. Inf. Dev. 29, 251–264. doi: 10.1177/0266666912458515

[ref53] Yan S. Zhang H. Wang J. (2022). Trends and hot topics in radiology, nuclear medicine and medical imaging from 2011–2021: a bibliometric analysis of highly cited papers. Jpn. J. Radiol. 40, 847–856. doi: 10.1007/s11604-022-01268-z, PMID: 35344133 PMC8958482

[ref54] Zhu H. Lei L. (2021). A dependency-based machine learning approach to the identification of research topics: a case in COVID-19 studies. Lib. Hi Tech 40, 495–515. doi: 10.1108/LHT-01-2021-0051

[ref55] Zhu H. Lei L. (2022). The research trends of text classification studies (2000–2020): a bibliometric analysis. SAGE Open 12, 215824402210899–215824402210816. doi: 10.1177%2F21582440221089963

